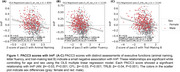# Plasma Levels of the Gut Bacteria‐Derived Metabolite Imidazole Propionate Are Negatively Associated with Cognitive Scores

**DOI:** 10.1002/alz70856_105650

**Published:** 2026-01-09

**Authors:** Jea Woo Kang, Vaibhav Vemuganti, Erin M. Jonaitis, Sterling C Johnson, Sanjay Asthana, Cynthia M. Carlsson, Nathaniel A. Chin, Corinne D. Engelman, Tyler K. Ulland, Federico E. Rey, Barbara B. Bendlin

**Affiliations:** ^1^ Wisconsin Alzheimer's Disease Research Center, University of Wisconsin School of Medicine and Public Health, Madison, WI, USA; ^2^ Department of Bacteriology, University of Wisconsin‐Madison, Madison, WI, USA; ^3^ Wisconsin Alzheimer's Disease Research Center, University of Wisconsin School of Medicine and Public Health, Madison, WI, USA; ^4^ Wisconsin Alzheimer's Disease Research Center, School of Medicine and Public Health, University of Wisconsin‐Madison, Madison, WI, USA; ^5^ Department of Population Health Sciences, University of Wisconsin School of Medicine and Public Health, Madison, WI, USA; ^6^ Department of Pathology and Laboratory Medicine, University of Wisconsin‐Madison, Madison, WI, USA; ^7^ Wisconsin Alzheimer's Disease Research Center, School of Medicine and Public Health, University of Wisconsin‐Madison, Madison, WI, USA

## Abstract

**Background:**

Among the various modifiable risk factors for AD development, the gut microbiome stands out as a potential therapeutic target, offering opportunities for intervention in the very early stages to potentially prevent disease onset. Notably, specific gut microbial metabolites may critically modulate metabolic and neuroimmune mechanisms shared with type 2 diabetes (T2D) and atherosclerosis—conditions that increase the risk of neurovascular and neurodegenerative disorders. Among these metabolites, imidazole propionate (ImP), a gut bacteria‐derived metabolite of histidine, has garnered attention for its potential to cross the blood‐brain barrier and exacerbate neurodegenerative processes.

**Method:**

Participants included in the analysis were from Wisconsin ADRC and Wisconsin Registry for Alzheimer's Prevention (WRAP) studies. ImP was determined using the Metabolon platform. Three composite tests for measuring executive functions, along with assessments of two other cognitive domains—immediate learning and delayed recall—were used to compute the global composite scores for the three‐test version of the Preclinical Alzheimer's Cognitive Composite (PACC3). The PACC3 scores were derived using three distinct measures of executive functions—Animal Naming Test (PACC3‐AN, *n* = 859), Category Fluency Test (PACC3‐CFL, *n* = 1118), and Trail‐Making Test B (PACC3‐TRLB, *n* = 1116)—and subsequently transformed into z‐scores. Ordinary Least Squares (OLS) multiple linear regression approach was used to evaluate the relationship between levels of ImP and cognitive scores while accounting for covariates such as age and sex in the analysis.

**Result:**

Lower cognitive performance was associated with higher levels of plasma ImP while controlling for age and sex, even in cognitively normal individuals before the onset of detectable cognitive symptoms.

**Conclusion:**

Despite the modest associations, the significance of predictors suggests these factors warrant further exploration in understanding their combined contribution to ImP levels and broader neurodegenerative mechanisms.